# Validation of the Coping with Health Injuries and Problems questionnaire in a longitudinal cohort with recent-onset RA

**DOI:** 10.1093/rap/rkaf057

**Published:** 2025-05-22

**Authors:** Zafer Akman, Gilles Boire, Nathalie Carrier, Sophie Roux, Ariel Masetto, Artur J de Brum-Fernandes, Patrick Liang, Hugues Allard-Chamard

**Affiliations:** Department of Medicine, Yale University School of Medicine, New Haven, CT, USA; Centre de recherche du Centre hospitalier universitaire de Sherbrooke (CRCHUS), Sherbrooke, QC, Canada; Department of Medicine, Division of Rheumatology, Faculty of Medicine and Health Sciences, Université de Sherbrooke, Sherbrooke, QC, Canada; Centre de recherche du Centre hospitalier universitaire de Sherbrooke (CRCHUS), Sherbrooke, QC, Canada; Centre de recherche du Centre hospitalier universitaire de Sherbrooke (CRCHUS), Sherbrooke, QC, Canada; Department of Medicine, Division of Rheumatology, Faculty of Medicine and Health Sciences, Université de Sherbrooke, Sherbrooke, QC, Canada; Centre de recherche du Centre hospitalier universitaire de Sherbrooke (CRCHUS), Sherbrooke, QC, Canada; Department of Medicine, Division of Rheumatology, Faculty of Medicine and Health Sciences, Université de Sherbrooke, Sherbrooke, QC, Canada; Centre de recherche du Centre hospitalier universitaire de Sherbrooke (CRCHUS), Sherbrooke, QC, Canada; Department of Medicine, Division of Rheumatology, Faculty of Medicine and Health Sciences, Université de Sherbrooke, Sherbrooke, QC, Canada; Centre de recherche du Centre hospitalier universitaire de Sherbrooke (CRCHUS), Sherbrooke, QC, Canada; Department of Medicine, Division of Rheumatology, Faculty of Medicine and Health Sciences, Université de Sherbrooke, Sherbrooke, QC, Canada; Centre de recherche du Centre hospitalier universitaire de Sherbrooke (CRCHUS), Sherbrooke, QC, Canada; Department of Medicine, Division of Rheumatology, Faculty of Medicine and Health Sciences, Université de Sherbrooke, Sherbrooke, QC, Canada

**Keywords:** coping factors, RA, patient experience, observational studies, outcome measures, wider determinants of health

## Abstract

**Objective:**

To validate the Coping with Health Injuries and Problems (CHIP) questionnaire in a prospective cohort of early RA patients.

**Methods:**

Between 2006 and 2022, newly diagnosed RA patients self-administered CHIP at baseline and at follow-up visits. The original CHIP comprises four subscales (Distraction, Palliative, Instrumental, Emotional preoccupation), each containing eight items (scores 8 to 40). At inclusion and again after more than 2 years of follow-up, internal consistency was assessed with Cronbach’s alpha, factor structure with exploratory and confirmatory factor analyses (EFA, CFA), and sensitivity to change with mixed linear models with repeated measures.

**Results:**

In 381 early RA patients, the means (SD) were 23.75 (6.52) for Distraction, 23.55 (6.11) for Palliative, 31.38 (5.41) for Instrumental and 25.11 (7.89) for Emotional preoccupation, values comparable to the literature only available in back pain patients. In 253 of the 381 patients followed up into established RA, all subscales except Distraction had decreased significantly between inclusion and follow-up. Internal consistency was similar in established and early RA (Cronbach’s alphas: 0.77 to 0.89 *vs* 0.75 to 0.86, respectively). EFA in early and established RA suggested that three items linked to treatment adherence consistently segregated from other Instrumental items as a subscale, although this did not improve internal consistency and CFA significantly.

**Conclusion:**

The original CHIP possesses good psychometric properties to describe individual coping styles in both early and established RA. Coping in RA might be better characterized using five rather than four subscales, with the additional subscale addressing treatment adherence.

Key messages

- CHIP possesses good psychometric properties to describe individual coping styles in both recent-onset and established RA.
- Coping in early RA may be better characterized using an additional subscale addressing treatment adherence.
- Emotional preoccupation, Instrumental and Palliative subscales significantly decreased between inclusion and long-term follow-up in 253 patients.

## Introduction

RA is a prevalent chronic systemic inflammatory disease [1, 2] with fluctuating manifestations [3]. Outcomes of RA patients, such as erosive joint damage and the need for surgery, have markedly improved over the recent decades [1, 2, 4]. Nonetheless, RA remains one of the world’s leading causes of disability and is associated with increased mortality and morbidity [5–8]. RA can lead to fatigue, physical limitations, sleep perturbations, emotional distress and poor quality of life. Consequently, patient-reported outcomes (PROs) remain severely impacted among those with recent-onset [9] as well as with established RA [10]. Persistent pain, non-adherence and defective coping all appear to play significant roles [11] in patients with poor outcomes.

Coping was defined, in 1984, as a dynamic and changing process engaged to manage internal and/or external stress, involving thoughts, behaviour or feelings [12, 13]. Coping may be general (reaction to any stressor) or specific (reaction to a specific stressor). The coping strategies employed by patients to face the emotional challenges of recent-onset and established RA could thus have a tremendous impact on their clinical outcomes.

Despite the importance of coping for RA, we currently lack validated tools to assess the coping mechanisms triggered by early RA and how the different coping styles impact disease evolution [14]. This missing tool may have contributed to the weak correlation of coping strategies with RA outcomes observed in early studies [15]. Several measures of coping were nonetheless recently used in RA despite little or no formal evidence of their ability to adequately assess coping strategies in RA. This is the case for versions of the Inventory for the Measurement of Coping Strategies with Stress (COPE) [16, 17], the Utrecht Coping list [9] and for some brief questionnaires [18, 19]. Although short questionnaires are more feasible in clinical practice, they typically address general resilience and not disease-related coping; they also do not provide as much information as longer questionnaires.

The CHIP version 5 [20] explores four domains of coping: Instrumental, Emotional preoccupation, Palliative and Distraction strategies. The Instrumental subscale is based on a person’s problem-solving inclination. Patients with a high Instrumental subscale tend to be involved in activities minimizing discomfort and related to recovery from the health problem, such as research for information about the disease and its treatment. The Emotional preoccupation subscale relates to the thoughts of the patient about their health-related problem. The patient may be in search of the causes of the health problem and of reasons why this disease is specifically affecting them. As a result, patients with a high score on the Emotional preoccupation subscale tend to be angry and anxious about health-related problems. The Palliative subscale assesses self-caring activities and efforts to improve one’s environment. A patient with a high Palliative score tends to minimize the negative consequences of health-related problems. The Distraction subscale assesses the use of pleasing experiences and involves activities unrelated to the health problem. Emotional preoccupation and Palliative coping have been linked to maladaptive behaviours, while Distraction and Instrumental coping are generally associated with adaptive outcomes.

CHIP has been shown to have good internal consistency with Cronbach’s alpha between 0.70 and 0.87 in numerous patient populations affected by pain, cancer, cardiac and psychiatric conditions [20]. Nonetheless, it has rarely been studied in inflammatory conditions, except for Inflammatory Bowel Disease [21], and was never validated per se in a rheumatological condition, such as RA. Our study is thus aimed at the validation of the CHIP questionnaire in a longitudinal cohort of patients with early RA, both at diagnosis and once the disease is well-established.

## Methods

### Cohort description

The longitudinal non-interventional Early Undifferentiated PolyArthritis (EUPA) cohort was previously described [22–26]. From July 1998 at the Centre hospitalier universitaire de Sherbrooke (CHUS; now CIUSSS de l’Estrie—CHUS), consecutive early inflammatory arthritis patients were invited to enrol. The inclusion criteria consisted of age 18 or older, inflammatory joint symptoms for at least 1 month and less than 1 year, and having at least three joints with synovitis at the time of inclusion. Patients were evaluated at baseline and at 12, 18, 30, 42 and 60 months after onset. Time of onset was defined as the self-reported week of first perception of inflammatory signs/symptoms. Treatment was left to the discretion of the rheumatologist and the patient, with the aim of remission. Only those patients fulfilling 1987/2010 criteria for RA within the first 18 months were retained for this analysis.

### Disease variables

A rheumatologist performed tender (TJC; 68 joints) and swollen (SJC; 66 joints) counts. A trained coordinator performed a structured interview at inclusion and each of the follow-up visits. Collected data included age, biological sex, BMI, symptom duration, previous and current medication, tobacco usage, alcohol consumption, ACR/EULAR criteria for RA and visual analogue scales (VAS; 0–100 mm) for patient (PtGA) and physician (EGA) Global Evaluation of Disease activity. Levels of CRP, IgM RF and anti-CCP2 antibody levels were assessed. SDAI scores were determined. Joint radiographs were reviewed by a trained reviewer (GB) according to the Sharp-van der Heijde (SvH) method, with a maximum score of 448 units [23, 27]. Collected data also included the Modified HAQ (M-HAQ) [28], Centre for Epidemiologic Studies—Depression (CES-D) [29], CHIP and VAS (0–100 mm) to measure pain levels and fatigue.

### The CHIP version 5

The CHIP questionnaire [21] is divided into four domains (or subscales): Instrumental, Emotional preoccupation, Palliative and Distraction strategies. It consists of a total of 32 items (Supplementary Table S1, available at *Rheumatology Advances in Practice* online) divided in four groups of eight. Participants rate each question on a 5-point scale (1 = not at all; 5 = very likely). The scores for each subscale thus range from 8 to 40. A higher score indicates that the patient uses more this particular coping strategy.

### Statistical analysis

For all validation analyses, a first analysis was performed on early RA patients and another on established RA. Early RA patients included only baseline CHIP questionnaires and established RA considered the first complete CHIP questionnaire filled by the patient after at least 30 months of disease duration.

The factor structure of CHIP was evaluated with exploratory factor analysis (EFA) with maximum likelihood (ML) estimation algorithms and the Promax rotation method with Kaiser Normalization. A first EFA without imposed number of factors (or subscales) was completed considering factors with an eigenvalue >1 and the scree curve was drawn to help define the optimal number of factors per model. The method of eigenvalue is acknowledged as the least accurate methods [30, 31]. To select the optimal number of factors, new models were conducted by manually selecting the number of factors based on scree curves. After rotation, only models with factors loading higher than 0.30, encompassing at least three items by factor and with limited cross loading, were retained [30, 31]. New models were also compared with the original structure for validation. Model fit was assessed with confirmatory factor analysis (CFA) with Maximum Likelihood with Robust standard errors (MLR) method and by the comparative fit index (CFI), normed fit index (NFI), standardized root mean squared residual (SRMR) and the root mean square error of approximation (RMSEA). CFI and NFI higher than 0.95, SRMR below 0.08 and RMSEA below 0.07 typically indicate a good model fit [32].

Internal consistency was measured with Cronbach’s alpha considering α adequate if higher than 0.70. Items were analysed considering corrected item-total correlations and a cut-off > 0.30 was adopted [33].

Sensitivity to change was examined through a linear mixed regression model considering visits from baseline up to 60 months of follow-up. The maximum-likelihood method and the variance components correlation matrix on the CHIP were used. Analyses were adjusted for sex, age, BMI and smoking effects and conducted with SAS version 9.4 (SAS Institute Inc, Cary, NC, USA). Race was not adjusted for, due to the homogeneity of our cohort. Figures were created on GraphPad Prism version 9.

### Ethical considerations

The study was approved and conducted in accordance with The Research Ethics Board of the CIUSSS de l’Estrie – CHUS (ClinicalTrials.gov ID: NCT00512239). Informed consent was obtained from each patient, and all signed a consent form in order to be included in EUPA.

## Results

### Cohort description

Between 8 June 2006 (the time of implementation of the CHIP questionnaire) and 6 October 2022, 612 patients were recruited in EUPA and 381 patients with complete baseline CHIP questionnaires were retained for analysis (Supplementary Fig. S1, available at *Rheumatology Advances in Practice* online). Of the of 231 excluded patients, 27 met exclusion criteria for EUPA over follow-up, and 27 did not fulfill RA criteria over the first 18 months of disease. The major reason for exclusion (177 patients) was absent (65) or incomplete (112) CHIP questionnaires at inclusion. In incomplete questionnaires, the distribution of missing answers appeared relatively random and at low prevalence (0.8–4.5%), the most frequently left unanswered being ‘Seek medical treatment as soon as possible’ (Supplementary Table S1, available at *Rheumatology Advances in Practice* online).

At baseline, median age was 60.5 years, 60% were women, median duration of symptoms was 4 months, and 14.4% were active smokers (Table 1). Patients had moderate to high disease activity, with a median SDAI of 29.4, 12 SJC, 12 TJC and elevated pain, depressive symptoms and fatigue. However, little radiographic damage was present. The baseline mean scores (SD) of the four CHIP subscales were 23.75 (6.52) for Distraction, 23.55 (6.11) for Palliative, 31.38 (5.41) for Instrumental and 25.11 (7.89) for Emotional preoccupation.

**Table 1.** Baseline patient characteristics

Variable	*N*	Value
Age (years)	381	60.5 (52.7–70.2)
Women, *n* (%)	381	228 (59.8)
BMI (kg/m^2^)	379	27 (23.5–30.3)
Tobacco, *n* (%)		
Current smoker	375	54 (14.4)
Ex-smoker		177 (47.2)
Non-smoker		144 (38.4)
Symptom duration (months)	380	4 (2.3–6.5)
Anti-CCP2 > 5 IU/ml, *n* (%)	379	134 (35.4)
RF ≥40 IU/ml	381	134 (35.2)
Any of RF/CCP2, *n* (%)	379	164 (43.3)
Erythrocyte sedimentation rate (ESR)	380	24 (13–44)
CRP	381	9.1 (3.3–27)
Modified HAQ (M-HAQ)	376	0.8 (0.4–1.3)
M-HAQ ≥ 1, *n* (%)	376	157 (41.8)
66-Swollen joint count	381	12 (7–19)
68-Tender joint count	379	12 (6–18)
Evaluator global assessment of disease activity (EGA) (0–100 mm)	377	4.7 (2.9–7)
Patient global assessment of disease activity (PtGA) (0–100 mm)	380	5.7 (3.8–7.8)
Pain (0–100 mm)	378	58 (32–76)
Fatigue (0–100 mm)	378	56 (35–76)
CES-D	350	17 (11–25)
DAS28-CRP	378	5.0 (4.1–6.1)
SDAI	375	29.4 (20.8–44.1)
CDAI	375	28.0 (19.6–39.7)
SvH total score	361	2 (0–6)
SvH erosion score	361	0 (0–2)
SvH narrowing score	362	1 (0–4)
CHIP questionnaire, mean (SD)		
Distraction	381	23.75 (6.52)
Palliative	381	23.55 (6.11)
Instrumental	381	31.38 (5.41)
Emotional preoccupation	381	25.11 (7.89)

Variables were presented with median (IQR: interquartile range) except when indicated *n* (%) or mean (SD). CES-D: Centre for Epidemiologic Studies—Depression; DAS28-CRP: 28-Joint Disease Activity Score Based on C-Reactive Protein; SDAI: Simplified Disease Activity Index; CDAI: Clinical Disease Activity Index; CHIP: Coping with Health Injuries and Problems; SvH: Sharp-van der Heijde Score.

Of the 381 patients with complete CHIP at baseline, 253 had at least one follow-up with a complete CHIP questionnaire at 30 (181 patients), 42 (56 patients) and 60 months (16 patients) and were considered at this time to have established disease. The 128 patients without a complete follow-up CHIP were similar at baseline to the 253 established RA patients who did contribute one, except they were older and less frequently seropositive and erosive (Supplementary Table S2, available at *Rheumatology Advances in Practice* online). Baseline patient characteristics were previously reported before and after COVID-19 pandemic and found to be similar [26]. Only 37 recruitments and 14 follow-ups with complete CHIP questionnaires were available between 15 March 2020 and 6 October 2022. No significant differences were present in scores of any CHIP categories obtained before and during the COVID pandemic at baseline and in established RA (Supplementary Table S3, available at *Rheumatology Advances in Practice* online). We were thus underpowered to study the impact of the COVID-19 pandemic on CHIP performance in our patients.

### The factor structure of CHIP in early RA

Using baseline values only, the EFA without a priori settings suggested an eight-subscale model with an eigenvalue >1 rather than the original four subscales. However, the eight-subscale model appeared suboptimal because subscales 7 and 8 were redundant, as they were defined by items already present in previous factors. In addition, subscale 8 was defined by only two items. For these reasons, and because scree curves (Supplementary Fig. S2A, available at *Rheumatology Advances in Practice* online) suggested that four to six factors may be optimal, this eight-subscale model was not retained for further analysis (Supplementary Table S4, available at *Rheumatology Advances in Practice* online).

An EFA in which we imposed four factors only was then computed. Rather than finding the four subscales of the original tool, we observed that three items addressing treatment adherence (i.e. items 19, 23 and 27) again segregated apart from other Instrumental items, while the Palliative items were distributed between the Distraction and Instrumental subscales (Supplementary Table S5, available at *Rheumatology Advances in Practice* online). In the EFA with imposed six factors, the three Adherence items again segregated apart, while the Palliative items were distributed between the Distraction and Instrumental subscales (Supplementary Table S6, available at *Rheumatology Advances in Practice* online). These results suggested that ‘Adherence’ items may represent a separate dimension in early RA.

We therefore performed an analysis with imposed five factors in total to account for the specificity of Adherence-related items and avoid scattering of pertinent clinical elements (Table 2). In this EFA, all items were distributed among the four subscales of the original tool, except for the three Adherence items segregating apart from other Instrumental items. This five-subscale structure accounted for 50% of the total variability of the CHIP questionnaires, compared with 45, 53 and 60% for the four-, six- and eight-subscale analyses, respectively.

**Table 2.** Results of exploratory factorial analysis with 5 imposed factors using the 32 coping items of the CHIP questionnaire in early RA (items found in the same dimension are shown in bold)

Items of the original CHIP grouped by subscales	Factor 1: Emotional preoccupation	Factor 2: Distraction	Factor 3: Instrumental	Factor 4: Adherence	Factor 5: Palliative
Emotional preoccupation					
4. Wonder “why me”	**0.649**	0.190	0.323	0.161	0.402
8. Feel angry	**0.664**	0.146	0.270	0.015	0.239
12. Become frustrated	**0.668**	0.057	0.243	0.003	0.270
16. Think about things I can’t do	**0.639**	0.101	0.324	0.202	0.270
20. Fantasize about being healthy	**0.580**	0.263	0.196	0.211	0.324
24. Wish it hadn’t happened	**0.591**	0.121	0.140	0.208	0.391
28. Think about being vulnerable	**0.713**	–0.056	0.221	0.088	0.394
32. Worry about my health	**0.778**	–0.047	0.225	0.142	0.289
Distraction					
1. Think about better times	0.149	**0.626**	0.200	0.196	0.254
5. Be with others	0.083	**0.578**	0.301	0.096	0.179
9. Daydream	–0.038	**0.691**	0.252	0.266	0.150
13. Enjoy attention from people	0.195	**0.468**	0.356	0.326	0.404
17. Plan for the future	0.150	**0.479**	0.407	0.130	0.221
21. Listen to music	0.067	**0.393**	0.302	0.147	0.309
25. Invite company	0.059	**0.531**	0.211	0.179	0.337
29. Have nice things around	0.053	**0.532**	0.332	0.269	0.491
Instrumental					
3. Find out more information	0.248	0.242	**0.675**	0.188	0.278
7. Seek treatment quickly	0.282	0.234	**0.446**	0.311	0.281
11. Focus on getting better	0.105	0.371	**0.589**	0.322	0.185
15. Learn more	0.255	0.348	**0.758**	0.192	0.365
31. Find out about treatment	0.306	0.298	**0.618**	0.334	0.405
19. Comply with advice	0.152	0.267	0.316	**0.827**	0.251
23. Follow doctor’s advice	0.147	0.292	0.308	**0.843**	0.335
27. Take medications on time	0.119	0.199	0.187	**0.549**	0.272
Palliative					
2. Stay in bed	0.384	0.077	0.239	–0.097	**0.395**
6. Rest when tired	0.366	0.202	0.317	0.115	**0.473**
10. Sleep	0.243	0.251	0.262	0.039	**0.363**
14. Conserve energy	0.432	0.105	0.292	0.115	**0.512**
18. Stay warm	0.262	0.385	0.230	0.321	**0.428**
22. Make surroundings quiet	0.251	0.368	0.329	0.296	**0.664**
26. Stay quiet	0.309	0.098	0.139	0.244	**0.609**
30. Get comfortable	0.149	0.454	0.315	0.413	**0.616**
Statistical summary of the scales					
Eigenvalues	7.209	3.414	1.983	1.716	1.538
% of variance explained	22.528	10.669	6.196	5.362	4.807
Cronbach's alpha	0.858	0.765	0.756	0.776	0.749

CFAs for the original four- and the potential five-subscale models were compared to evaluate which structure represented the best model fit (Table 3). The five-subscale structure had better fit values for all measures. This suggested that if changes were to be made to analyses of CHIP when used in early RA, the three Adherence items could be analysed separately as a subscale or domain for enhanced perspective on the coping strategies used by patients.

**Table 3.** Confirmatory analysis of the CHIP questionnaire in early RA

Model	X^2^	Df	*P*-value	CFI	NFI	SRMR	RMSEA
4 factors	1518.5618	458	<0.0001	0.7284	0.6549	0.0781	0.0781
5 factors	1247.6773	454	<0.0001	0.7967	0.7165	0.0732	0.0678
6 factors	931.2956	390	<0.0001	0.8496	0.7691	0.0673	0.0604

Df: degree of freedom; CFI: comparative fit index; NFI: normed fit index; SRMR: standardized root mean squared residual; RMSEA: root mean square error of approximation.

### Internal consistency in early RA

The analysis of internal consistency of CHIP questionnaires with the original related individual subscales is shown in Table 4. Cronbach’s alpha revealed adequate internal consistency for Emotional preoccupation (0.86), Instrumental (0.77), Distraction (0.77) and Palliative (0.75) dimensions. Deleting each element separately did not improve the coefficient, and all items had corrected item-total correlations > 0.30, suggesting that all items had good correlation with their respective subscale. Cronbach’s alphas on the split dimensions in the five-factor structure evaluated above were not improved for the remaining five items in the Instrumental Dimension (0.76) nor the three isolated Adherence items (0.75). Although factor analysis suggested that coping in early disease may be better characterized using five rather than four subscales, analysis of internal consistency suggested that using the original four-subscale version might be comparable. Therefore, we used the original four-subscale structure of CHIP in the following Sensitivity to change analysis.

**Table 4.** Internal consistency as measured by Cronbach’s alpha in early RA if each item was deleted one at a time and by corrected item-total correlations

	Score Mean ± SD	Corrected item-total correlation	Cronbach's alpha if item deleted
Distraction (Cronbach alpha = 0.77)			
1. Think about better times	2.98 ± 1.34	0.498	0.734
5. Be with others	2.78 ± 1.27	0.497	0.735
9. Daydream	3.12 ± 1.31	0.541	0.727
13. Enjoy attention from people	3.72 ± 1.24	0.432	0.746
17. Plan for the future	2.83 ± 1.33	0.426	0.747
21. Listen to music	3.08 ± 1.39	0.358	0.760
25. Invite company	2.33 ± 1.25	0.486	0.737
29. Have nice things around	2.92 ± 1.46	0.484	0.737
Palliative (Cronbach alpha = 0.75)			
2. Stay in bed	1.99 ± 1.27	0.373	0.736
6. Rest when tired	3.30 ± 1.34	0.525	0.707
10. Sleep	2.67 ± 1.28	0.386	0.734
14. Conserve energy	2.67 ± 1.21	0.488	0.715
18. Stay warm	3.34 ± 1.36	0.393	0.733
22. Make surroundings quiet	3.27 ± 1.27	0.510	0.710
26. Stay quiet	2.64 ± 1.29	0.457	0.720
30. Get comfortable	3.67 ± 1.10	0.427	0.727
Instrumental (Cronbach alpha = 0.77)			
3. Find out more information	3.41 ± 1.33	0.530	0.739
7. Seek treatment quickly	3.63 ± 1.27	0.432	0.758
11. Focus on getting better	3.62 ± 1.15	0.513	0.741
15. Learn more	3.17 ± 1.25	0.544	0.735
19. Comply with advice	4.56 ± 0.71	0.489	0.752
23. Follow doctor’s advice	4.56 ± 0.73	0.487	0.752
27. Take medications on time	4.62 ± 0.81	0.318	0.771
31. Find out about treatment	3.80 ± 1.23	0.556	0.733
Emotional preoccupation (Cronbach alpha = 0.86)			
4. Wonder “why me”	2.93 ± 1.52	0.602	0.842
8. Feel angry	1.91 ± 1.23	0.614	0.841
12. Become frustrated	2.66 ± 1.42	0.607	0.841
16. Think about things I can’t do	3.33 ± 1.36	0.587	0.843
20. Fantasize about being healthy	3.44 ± 1.45	0.549	0.848
24. Wish it hadn’t happened	3.87 ± 1.42	0.559	0.846
28. Think about being vulnerable	3.17 ± 1.44	0.612	0.840
32. Worry about my health	3.80 ± 1.27	0.712	0.830

Cronbach’s alpha was not improved when the Instrumental scale was divided into two subscales: Adherence (Items 19, 23 and 27, alpha=0.76) and instrumental without adherence items (alpha=0.75).

### Sensitivity to change in early RA followed up over 5 years

**Figure 1.**
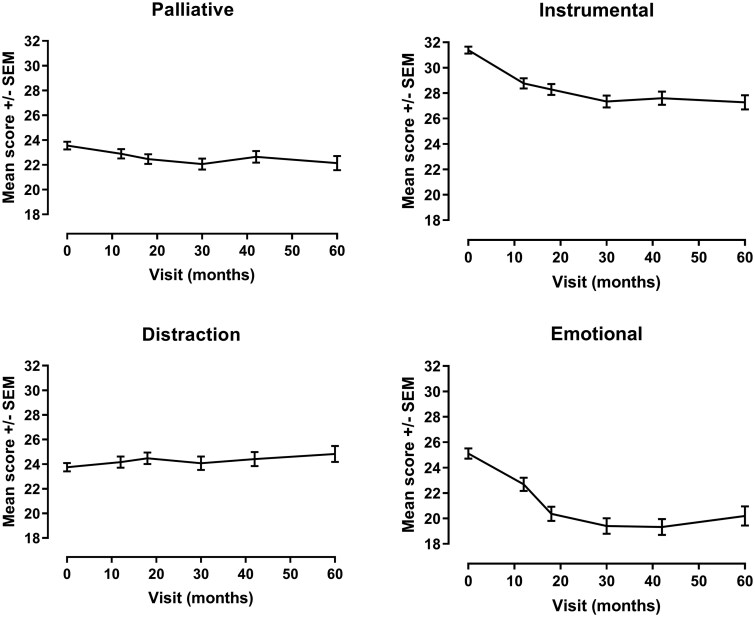
Sensitivity to change of CHIP dimensions in early RA followed up over 5 years. CHIP: Coping with Health Injuries and Problems

As shown in Fig. 1, dimensions of the CHIP scale were sensitive to change with a significant decrease in mean scores [± standard error of the mean (SEM)] over 5 years for Instrumental (−4.11 ± 0.62, *P* < 0.001), Emotional preoccupation (−4.91 ± 0.82, *P* < 0.001) and a trend for Palliative (−1.41 ± 0.61, *P* = 0.06) subscales. No change was observed for Distraction (1.07 ± 0.71, *P* = 0.7).

After adjustment for age, sex, BMI and smoking status at baseline, changes in Distraction remained nonsignificant; decreases in Instrumental (−4.04 ± 0.60, *P* < 0.001) and Emotional preoccupation (−5.00 ± 0.79, *P* < 0.001) remained significant and those in Palliative became significant (−1.48 ± 0.43, *P* = 0.001). This implies that Emotional preoccupation, Instrumental and Palliative coping strategies were more used in early disease and their use decreased over time, plateauing at 30 months.

### Factor structure and internal consistency in patients with established RA

Our early RA patients were followed longitudinally and evolved gradually into ‘established RA’. We analysed the 253 patients with at least one complete CHIP questionnaire both at baseline and after at least 30 months of follow-up. We performed Factor structure and Internal consistency analyses in these 253 patients using their first complete questionnaire available at their 30-month visit or later. The scree curve was very similar to the one obtained with early RA (Supplementary Fig. S2B, available at *Rheumatology Advances in Practice* online). The EFA without a priori settings suggested a model with seven subscales with an eigenvalue >1 but was suboptimal because all 32 items were already defined in the first six subscales; subscale 7 was thus redundant. When we tried to impose four or five factors, the adherence items segregated from other Instrumental items as they did in early RA, but Palliative items were distributed in the Emotional and Distraction subscales. This suggested that a six-subscale model might be better. EFA results for six imposed factors are thus presented in Table 5. These six subscales accounted for 60% of total variability, versus 51, 56, 63 and 66% for the four-, five-, seven- and eight-factor models. In the six-factor model for established RA, the three Adherence-related items (items 19, 23 and 27) segregated again as a subscale apart from other Instrumental items. However, items of the Palliative dimension were then split to constitute the two last subscales containing items 2, 6, 10, 14 and items 14, 18, 22, 26, 30, with cross loading of item 14, now present in two subscales.

**Table 5.** Results of exploratory factorial analysis with imposed 6 factors using the 32 coping items of the CHIP questionnaire in established RA (items found in the same dimension are shown in bold)

Items of the original CHIP grouped by subscales	Factor 1:	Factor 2:	Factor 3:	Factor 4:	Factor 5:	Factor 6:
	Emotional preoccupation	Distraction	Adherence	Instrumental	Rest	Comforts
Emotional preoccupation						
4. Wonder “why me”	**0.731**	0.030	0.054	0.458	0.384	0.186
8. Feel angry	**0.588**	0.001	–0.069	0.267	0.300	0.258
12. Become frustrated	**0.753**	–0.048	–0.014	0.299	0.391	0.223
16. Think about things I can’t do	**0.769**	0.084	0.059	0.479	0.456	0.368
20. Fantasize about being healthy	**0.705**	0.227	0.056	0.403	0.473	0.417
24. Wish it hadn’t happened	**0.666**	0.084	0.110	0.290	0.202	0.341
28. Think about being vulnerable	**0.777**	0.054	0.065	0.387	0.412	0.455
32. Worry about my health	**0.775**	0.050	0.191	0.386	0.317	0.289
Distraction						
1. Think about better times	0.111	**0.593**	0.191	0.217	0.316	0.014
5. Be with others	–0.012	**0.582**	0.193	0.312	0.071	0.077
9. Daydream	–0.034	**0.775**	0.275	0.274	0.110	0.101
13. Enjoy attention from people	0.214	**0.603**	0.333	0.316	0.172	0.318
17. Plan for the future	0.049	**0.525**	0.095	0.363	0.111	0.162
21. Listen to music	–0.012	**0.505**	0.118	0.231	0.020	0.198
25. Invite company	0.029	**0.596**	0.096	0.184	–0.001	0.251
29. Have nice things around	0.099	**0.703**	0.266	0.252	0.084	0.449
Instrumental						
19. Comply with advice	0.129	0.312	**0.878**	0.317	0.149	0.268
23. Follow doctor’s advice	0.035	0.310	**0.893**	0.186	0.080	0.221
27. Take medications on time	0.071	0.215	**0.728**	0.152	0.048	0.190
3. Find out more information	0.368	0.242	0.169	**0.725**	0.260	0.083
7. Seek treatment quickly	0.350	0.195	0.239	**0.536**	0.312	0.243
11. Focus on getting better	0.283	0.345	0.223	**0.657**	0.242	0.253
15. Learn more	0.330	0.408	0.165	**0.744**	0.162	0.452
31. Find out about treatment	0.526	0.337	0.150	**0.715**	0.238	0.415
Palliative						
2. Stay in bed	0.384	0.021	–0.011	0.139	**0.657**	0.244
6. Rest when tired	0.441	0.130	0.135	0.325	**0.668**	0.271
10. Sleep	0.315	0.227	0.162	0.310	**0.703**	0.220
14. Conserve energy	0.541	0.103	0.070	0.384	**0.550**	**0.570**
18. Stay warm	0.305	0.495	0.318	0.415	0.173	**0.488**
22. Make surroundings quiet	0.240	0.450	0.235	0.328	0.227	**0.549**
26. Stay quiet	0.433	0.129	0.162	0.209	0.318	**0.517**
30. Get comfortable	0.107	0.641	0.419	0.278	0.125	**0.555**
Statistical summary of the scales						
Eigenvalues	8.171	4.410	2.148	1.708	1.492	1.158
% of variance explained	25.534	13.782	6.711	5.337	4.663	3.619
Cronbach's alpha	0.893	0.824	0.870	0.799	0.718	0.720

Cronbach’s alpha values reflecting internal consistency in the original four-subscale CHIP were 0.82 for Distraction, 0.77 for Palliative, 0.79 for Instrumental and 0.89 for the Emotional preoccupation. Internal consistency of the Instrumental domain was improved in the six-subscale model in established disease by splitting the three items addressing Adherence (0.87) from the five remaining Instrumental items (0.80). On the contrary, Cronbach’s alphas slightly deteriorated when the Palliative subscale (0.75 in the original CHIP in early RA) was divided in the six-subscale model of established disease (0.72 and 0.73). In summary, the five-subscale model failed to give a good segregation of the Palliative factors but improved internal consistency, while the six-subscale model segregated the Palliative subscale but with lower Cronbach alpha values. Taking these information into account, we suggest the five-factor model defined in early RA might also be optimal in established RA, as it segregated the Adherence items and kept the items from the other subscales together.

## Discussion

The onset of persistent painful joint inflammation and systemic symptoms preceding RA diagnosis represents a very significant psychological and physical stressor from a patient perspective. We have shown in a large prospective cohort of early RA followed for a median of more than 4 years that the CHIP questionnaire appropriately describes individual coping styles in newly diagnosed patients as well as in patients with established RA. This fills a significant gap in the toolkit available for physicians to analyse patients’ variables contributing to short and long-term outcomes in RA.

There are very little data to compare our mean values for Distraction, Palliative, Instrumental and Emotional preoccupation subscales (23.75, 23.55, 31.38, 25.11, respectively) in comparable populations. For example, Flett (2011) did not report CHIP means in their inflammatory bowel disease patients. Further, direct comparisons with those of Endler *et al.* (1994) in back pain patients should be avoided as separate means for men (21.60, 24.43, 31.56, and 28.76) and women (23.64, 25.48, 33.78, and 29.41) were reported.

The four-subscale CHIP showed an adequate internal consistency in our cohort of early RA, similar to that observed in the sub-cohort of patients with low-back pain presented in the original CHIP manuscript [34]. Interestingly, EFAs at baseline suggested that questions addressing patients’ perceptions about treatment adherence may have intrinsic interrelations that segregated them from the other five questions of the Instrumental dimension. This underlines the importance of patient adherence in a disease for which highly effective (if taken) treatments are available. However, segregating these three questions to develop a five-subscale variant of CHIP did not improve internal consistency, but it slightly improved the fit of the overall model. Additionally, the five-subscale variant of the CHIP did not perform significantly better to identify the variability present in the data. Nonetheless, because it potentially contributes additional pertinent information about treatment adherence, we recommend using a modified five-factor CHIP questionnaire in both early and established RA.

CHIP appears to perform slightly better in established RA, where it showed better internal consistency than in recent-onset patients. Interestingly, the same three questions related to Adherence within the Instrumental dimension demonstrating unique psychometric properties in early RA, also stood out in EFAs in established RA. As adherence in chronic diseases such as RA remains quite difficult to assess [35, 36] and to improve, the input provided by these three questions may be worth exploring in future studies.

CHIP also demonstrated an excellent sensitivity to change with significant decreases from baseline to established disease in Emotional preoccupation, Instrumental and Palliative dimensions. This decrease in the use of coping strategies paralleled the decrease in disease activity and in depressive symptoms that we previously reported in our cohort [23]. Only the Distraction dimension did not change between baseline and follow-up. The decrease in Instrumental coping in established disease suggests that early RA patients are more prone to actively look for information about RA and its treatment relative to when their then established RA is generally controlled. Similarly, decreases in anger and anxiety and improvements in well-being once established RA is generally well controlled may explain the significant decreases in Emotional preoccupation and Palliative strategies.

Our study has numerous strengths. First, we used a large cohort of patients with recent-onset RA. Second, these patients were followed for several years, also allowing the study of CHIP in patients with established RA. Third, the prospective nature of our study allowed evaluation of the sensitivity to change of the questionnaire. Coping is very dynamic and personal when facing a new stressor, although dimensions such as Distraction behaviour strategy appear more static than the other dimensions, in the absence of specific interventions.

Our study also has limitations. First, validation of the CHIP tool being a secondary objective in our cohort, we did not plan to evaluate the test-retest validity over short periods of observation; this was previously evaluated and shown to be valid [34]. Second, in the absence of a gold standard for coping assessment in RA, we could not evaluate the construct validity by comparing CHIP’s properties with a validated measure of coping.

In summary, the CHIP questionnaire provides valuable measures of emotional and task-oriented responses to RA, both in early and in established disease. While addressing unfavourable coping skills cannot cure RA, it may represent a way to improve overall well-being, cultivate resilience and allow patients to lead fulfilling lives, despite the challenges posed by their condition. Indeed, we previously showed that mindfulness-based interventions in symptomatic RA patients in which disease activity appeared controlled led to improved stress and depression, as well as improved function [37].

## Conclusion

Our work in a large longitudinal cohort of patients with early RA followed for a median of 4.6 years confirms that the CHIP questionnaire may be a valid tool to assess coping strategies both in early and in established RA. Information provided by CHIP to the clinician regarding the patient’s perspective might be used clinically and orient towards early personalized interventions. Whether the questionnaire may also be used as a predictor of outcomes remains to be explored.

## Supplementary Material

rkaf057_Supplementary_Data

## Data Availability

The data underlying this article are available upon request to the corresponding author.
