# Supplementary material for: Validation of the Coping with Health Injuries and Problems questionnaire in a longitudinal cohort with recent-onset RA
Source: Rheumatol Adv Pract. 2025 May 22;9(3):rkaf057. doi: 10.1093/rap/rkaf057 (PMC12274643; doi:10.1093/rap/rkaf057)
Supplement: rkaf057_Supplementary_Data [file rkaf057_supplementary_data.docx]

612 EUPA patients recruited after the implementation of the CHIP questionnaire

**Early RA**

381 patients analysed at baseline

- 27 did not fulfill EUPA inclusion criteria or met exclusion criteria
- 27 did not fulfill 1987 or 2010 criteria for RA
- 65 did not complete CHIP at baseline
- 112 filled incomplete CHIP at baseline

**Established RA**

253 patients analysed after at least 30 months follow up

181 at 30 months

56 at 42 months

16 at 60 months

- 2 died
- 16 lost to follow up
- 34 withdrew consent
- 8 removed by research team
- 46 had less than 30 months of follow up
- 22 did not provide any complete CHIP questionnaire during follow up

**Supplementary Figure S1. Flowchart of EUPA patients recruited between June 8 2006 and October 6 2022, and available for analysis at baseline (Early RA) and after at least 30 months of follow up (Established RA).**

**Supplementary Figure S2. Scree curves from exploratory factorial analysis for early RA patients at baseline and in established RA patients**

**Supplementary Table S1. Distribution of missing data in CHIP questionnaires at baseline in 493 consecutive early RA patients (the most frequently missing item is shown in bold).**

|  | Missing data | |
| --- | --- | --- |
|  | n | % |
| **Distraction** |  |  |
| 1.     Think about the good time I’ve had. | 11 | 2.2 |
| 5.     Be with other people. | 15 | 3.0 |
| 9.     Daydream about pleasant things. | 14 | 2.8 |
| 13.  Enjoy the attention of friends and family. | 16 | 3.2 |
| 17.  Make plans for the future. | 9 | 1.8 |
| 21.  Listen to music. | 10 | 2.0 |
| 25.  Invite people to visit me. | 13 | 2.6 |
| 29.  Surround myself with nice things (e.g., flowers) | 11 | 2.2 |
| **Palliative** |  |  |
| 2.     Stay in bed. | 9 | 1.8 |
| 6.     Lie down when I feel tired. | 14 | 2.8 |
| 10.  Get plenty of sleep. | 15 | 3.0 |
| 14.  Try to use as little energy as possible. | 13 | 2.6 |
| 18.  Make sure I am warmly dressed or covered. | 12 | 2.4 |
| 22.  Make my surroundings as quiet as possible. | 7 | 1.4 |
| 26.  Be as quiet and still as I can. | 10 | 2.0 |
| 30.  Make sure I am comfortable. | 6 | 1.2 |
| **Instrumental** |  |  |
| 3.     Find out more information about the illness. | 8 | 1.6 |
| **7.     Seek medical treatment as soon as possible.** | **22** | **4.5** |
| 11.  Concentrate on the goal of getting better. | 14 | 2.8 |
| 15.  Learn more about how my body works. | 6 | 1.2 |
| 19.  Do what my doctor tells me. | 11 | 2.2 |
| 23.  Try my best to follow my doctor’s advice. | 4 | 0.8 |
| 27.  Be prompt about taking medications. | 6 | 1.2 |
| 31.  Learn more about the most effective treatments available. | 7 | 1.4 |
| **Emotional** |  |  |
| 4.     Wonder why it happened to me. | 8 | 1.6 |
| 8.     Become angry because it happened to me. | 11 | 2.2 |
| 12.  Get frustrated. | 9 | 1.8 |
| 16.  Feel anxious about the things I can’ do. | 11 | 2.2 |
| 20.  Fantasize about all things I could do if I was better. | 10 | 2.0 |
| 24.  Wish that the problem had never happened. | 9 | 1.8 |
| 28.  Feel anxious about being weak and vulnerable. | 12 | 2.4 |
| 32.  Worry that my health might get worse. | 6 | 1.2 |

**Supplementary Table S2. Baseline patient characteristics between the 253 analyzed patients with established RA and the 128 patients excluded because they did not complete a CHIP questionnaire after at least 30 months of follow-up**

|  | Excluded  (n=128) | Established  (n=253) | p-value |
| --- | --- | --- | --- |
| Age (years) | 65.3 (56.1-74.2) | 59.6 (51.1-68.9) | **0.004** |
| Women, n (%) | 80 (62.5) | 148 (58.5) | 0.452 |
| Body mass index (kg/m^2^) | 27.1 (23.8-30.3) | 27 (23.2-30.3) | 0.726 |
| Tobacco, n (%) |  |  |  |
| Current smoker | 20 (16.1) | 34 (13.5) | 0.581 |
| Ex-smoker | 54 (43.5) | 123 (49) |  |
| Non-smoker | 50 (40.3) | 94 (37.5) |  |
| Symptom duration (months) | 3.8 (2.3-5.8) | 4.1 (2.3-7.1) | 0.224 |
| Anti-CCP2 > 5 IU/ml, n (%) | 34 (26.6) | 100 (39.8) | **0.011** |
| Rheumatoid Factor ≥40 IU/ml | 40 (31.3) | 94 (37.2) | 0.254 |
| Erythrocyte sedimentation rate (ESR) | 24 (13.3-44) | 24 (13-44) | 0.603 |
| C-reactive protein (CRP) | 9 (4.6-23.9) | 9.3 (1-28.7) | 0.758 |
| Modified Health Assessment Questionnaire (M-HAQ) | 0.8 (0.4-1.4) | 0.8 (0.4-1.3) | 0.604 |
| 66-Swollen joint count | 12.5 (8-19) | 11 (7-18) | 0.095 |
| 68-Tender joint count | 12 (5-21) | 12 (6-17) | 0.247 |
| Patient global assessment of disease activity (PtGA)  (0-100 cm) | 6.2 (4.2-8) | 5.5 (3.2-7.8) | 0.066 |
| Pain (0-100 mm) | 62 (34-79) | 54 (31-74) | 0.153 |
| Fatigue (0-100 mm) | 60 (40-76) | 54 (33-76) | 0.180 |
| CES-D | 16 (11-24) | 18 (11-25) | 0.295 |
| Sharp van der Heijde (SvH) Total score | 2 (0-6) | 2 (1-6) | 0.242 |
| SvH erosion score | 0 (0-2) | 1 (0-2) | **0.002** |
| SvH narrowing score | 1 (0-4.3) | 1 (0-4) | 0.805 |

Variables were presented with median (IQR: Interquartile range) except when indicated n (%). Significant p values are shown in bold.

**Supplementary Table S3. CHIP score in early RA patients and in established RA patients before (June 8 2006 to March 14 2020) and during the COVID-19 pandemic (March 15 2020 to October 6 2022).**

|  | Before COVID-19 | During COVID-19 | p-value |
| --- | --- | --- | --- |
| Early RA (baseline) | (n=344) | (n=37) |  |
| Distraction | 23.90 ± 6.53 | 22.43 ± 6.35 | 0.150 |
| Palliative | 23.61 ± 6.14 | 22.92 ± 5.84 | 0.319 |
| Instrumental | 31.48 ± 5.42 | 30.38 ± 5.28 | 0.151 |
| Emotional Preoccupation | 25.22 ± 7.95 | 24.14 ± 7.35 | 0.359 |
| Established RA | (n=239) | (n=14) |  |
| Distraction | 24.54 ± 7.1 | 23.86 ± 6.46 | 0.808 |
| Palliative | 22.31 ± 5.93 | 23.93 ± 5.80 | 0.257 |
| Instrumental | 27.43 ± 6.09 | 28.86 ± 5.67 | 0.443 |
| Emotional Preoccupation | 19.71 ± 8.34 | 20.36 ± 7.40 | 0.655 |

**Supplementary Table S4. Results of exploratory factorial analysis without any imposed number of factors using the 32 coping items of the CHIP questionnaire in early RA (items found in the same dimension are shown in bold).**

| **Items of the original CHIP grouped by subscales** | **Factor 1:** | **Factor 2:** | **Factor 3:** | **Factor 4:** | **Factor 5:** | **Factor 6:** | **Factor 7:** | **Factor 8:** |
| --- | --- | --- | --- | --- | --- | --- | --- | --- |
|  | **Emotional preoccupation** | **Distraction** | **Instrumental** | **Adherence** | **Rest** | **Comforts** | **Anger** | **Worry** |
| Emotional preoccupation |  |  |  |  |  |  |  |  |
| 4. Wonder “why me” | **0.597** | 0.099 | 0.300 | 0.206 | 0.483 | 0.290 | **0.496** | 0.213 |
| 8. Feel angry | **0.509** | 0.092 | 0.252 | 0.053 | 0.326 | 0.164 | **0.775** | 0.328 |
| 12. Become frustrated | **0.516** | 0.011 | 0.226 | 0.047 | 0.353 | 0.148 | **0.595** | 0.415 |
| 16. Think about things I can’t do | **0.665** | 0.068 | 0.334 | 0.239 | 0.350 | 0.137 | 0.229 | 0.300 |
| 20. Fantasize about being healthy | **0.675** | 0.204 | 0.215 | 0.212 | 0.285 | 0.292 | 0.193 | 0.174 |
| 24. Wish it hadn’t happed | **0.655** | 0.023 | 0.142 | 0.226 | 0.363 | 0.318 | 0.237 | 0.211 |
| 28. Think about being vulnerable | **0.592** | -0.100 | 0.207 | 0.133 | 0.457 | 0.195 | 0.376 | **0.905** |
| 32. Worry about my health | **0.731** | -0.093 | 0.239 | 0.169 | 0.327 | 0.147 | 0.337 | **0.564** |
| Distraction |  |  |  |  |  |  |  |  |
| 1. Think about better times | 0.210 | **0.626** | 0.199 | 0.183 | 0.159 | 0.339 | 0.064 | -0.067 |
| 5. Be with others | 0.062 | **0.593** | 0.278 | 0.114 | 0.249 | 0.217 | 0.167 | -0.120 |
| 9. Daydream | 0.066 | **0.758** | 0.255 | 0.262 | 0.100 | 0.260 | -0.122 | -0.204 |
| 13. Enjoy attention from people | 0.301 | **0.409** | 0.353 | 0.334 | 0.324 | **0.414** | -0.005 | -0.049 |
| 17. Plan for the future | 0.178 | **0.458** | 0.404 | 0.138 | 0.195 | 0.276 | 0.105 | -0.015 |
| 21. Listen to music | 0.104 | **0.345** | 0.290 | 0.147 | 0.202 | 0.351 | 0.067 | -0.058 |
| 25. Invite company | 0.117 | **0.455** | 0.198 | 0.167 | 0.190 | **0.453** | 0.146 | -0.165 |
| 29. Have nice things around | 0.121 | **0.435** | 0.330 | 0.239 | 0.178 | **0.686** | 0.072 | -0.052 |
| Instrumental |  |  |  |  |  |  |  |  |
| 3. Find out more information | 0.228 | 0.199 | **0.671** | 0.223 | 0.284 | 0.245 | 0.171 | 0.091 |
| 7. Seek treatment quickly | 0.320 | 0.199 | **0.443** | 0.353 | 0.347 | 0.198 | 0.087 | 0.064 |
| 11. Focus on getting better | 0.194 | 0.370 | **0.609** | 0.349 | 0.182 | 0.200 | -0.066 | -0.067 |
| 15. Learn more | 0.248 | 0.304 | **0.740** | 0.236 | 0.339 | 0.324 | 0.181 | 0.067 |
| 31. Find out about treatment | 0.381 | 0.188 | **0.665** | 0.335 | 0.211 | 0.465 | 0.145 | 0.055 |
| 19. Comply with advice | 0.290 | 0.223 | 0.333 | **0.877** | 0.166 | 0.273 | -0.085 | -0.067 |
| 23. Follow doctor’s advice | 0.291 | 0.220 | 0.329 | **0.820** | 0.145 | 0.400 | -0.098 | -0.023 |
| 27. Take medications on time | 0.236 | 0.156 | 0.198 | **0.538** | 0.145 | 0.300 | -0.141 | 0.028 |
| Palliative |  |  |  |  |  |  |  |  |
| 2. Stay in bed | 0.264 | 0.030 | 0.189 | -0.035 | **0.557** | 0.217 | 0.344 | 0.267 |
| 6. Rest when tired | 0.367 | 0.161 | 0.272 | 0.195 | **0.722** | 0.267 | 0.133 | 0.141 |
| 10. Sleep | 0.194 | 0.240 | 0.219 | 0.091 | **0.516** | 0.243 | 0.141 | 0.140 |
| 14. Conserve energy | 0.455 | 0.036 | 0.273 | 0.166 | **0.544** | 0.319 | 0.131 | 0.236 |
| 18. Stay warm | 0.391 | 0.316 | 0.220 | 0.340 | 0.371 | **0.392** | 0.005 | -0.034 |
| 22. Make surroundings quiet | 0.329 | 0.268 | 0.306 | 0.314 | 0.450 | **0.598** | 0.031 | 0.125 |
| 26. Stay quiet | 0.355 | 0.001 | 0.120 | 0.261 | 0.425 | **0.475** | 0.053 | 0.239 |
| 30. Get comfortable | 0.313 | 0.318 | 0.314 | 0.395 | 0.302 | **0.741** | -0.045 | -0.090 |
| Statistical summary of the scales |  |  |  |  |  |  |  |  |
| Eigenvalues | 7.209 | 3.414 | 1.983 | 1.716 | 1.538 | 1.244 | 1.049 | 1.001 |
| % of variance explained | 22.528 | 10.669 | 6.196 | 5.362 | 4.807 | 3.886 | 3.278 | 3.127 |

**Supplementary Table S5. Results of exploratory factorial analysis with imposed 4 factors using the 32 coping items of the CHIP questionnaire in early RA (items found in the same dimension are shown in bold).**

| **Items of the original CHIP grouped by subscales** | **Factor 1:** | **Factor 2:** | **Factor 3:** | **Factor 4:** |
| --- | --- | --- | --- | --- |
|  | **Emotional preoccupation** | **Distraction** | **Adherence** | **Instrumental** |
| Emotional preoccupation |  |  |  |  |
| 4. Wonder “why me” | **0.657** | 0.274 | 0.212 | 0.288 |
| 8. Feel angry | **0.630** | 0.164 | 0.061 | 0.253 |
| 12. Become frustrated | **0.645** | 0.104 | 0.055 | 0.221 |
| 16. Think about things I can’t do | **0.622** | 0.138 | 0.242 | 0.308 |
| 20. Fantasize about being healthy | **0.573** | 0.287 | 0.244 | 0.179 |
| 24. Wish it hadn’t happed | **0.609** | 0.225 | 0.258 | 0.095 |
| 28. Think about being vulnerable | **0.722** | 0.072 | 0.158 | 0.175 |
| 32. Worry about my health | **0.757** | 0.025 | 0.203 | 0.197 |
| Distraction |  |  |  |  |
| 1. Think about better times | 0.169 | **0.529** | 0.185 | 0.210 |
| 5. Be with others | 0.097 | **0.487** | 0.084 | 0.309 |
| 9. Daydream | -0.015 | **0.539** | 0.228 | 0.274 |
| 13. Enjoy attention from people | 0.251 | **0.519** | 0.335 | 0.331 |
| 17. Plan for the future | 0.169 | **0.446** | 0.124 | 0.409 |
| 21. Listen to music | 0.119 | **0.432** | 0.153 | 0.279 |
| 25. Invite company | 0.112 | **0.557** | 0.177 | 0.186 |
| 29. Have nice things around | 0.144 | **0.628** | 0.277 | 0.288 |
| Instrumental |  |  |  |  |
| 3. Find out more information | 0.276 | 0.290 | 0.202 | **0.667** |
| 7. Seek treatment quickly | 0.306 | 0.276 | 0.326 | **0.437** |
| 11. Focus on getting better | 0.124 | 0.345 | 0.308 | **0.607** |
| 15. Learn more | 0.298 | 0.403 | 0.207 | **0.740** |
| 31. Find out about treatment | 0.353 | 0.389 | 0.356 | **0.591** |
| 19. Comply with advice | 0.183 | 0.295 | **0.816** | 0.309 |
| 23. Follow doctor’s advice | 0.197 | 0.356 | **0.848** | 0.287 |
| 27. Take medications on time | 0.163 | 0.260 | **0.555** | 0.165 |
| Palliative |  |  |  |  |
| 2. Stay in bed | **0.426** | 0.212 | -0.043 | 0.184 |
| 6. Rest when tired | **0.425** | 0.340 | 0.160 | 0.262 |
| 10. Sleep | 0.293 | **0.336** | 0.069 | 0.223 |
| 14. Conserve energy | **0.491** | 0.279 | 0.170 | 0.230 |
| 18. Stay warm | 0.316 | **0.462** | 0.340 | 0.195 |
| 22. Make surroundings quiet | 0.357 | **0.545** | 0.333 | 0.260 |
| 26. Stay quiet | **0.398** | 0.321 | 0.294 | 0.070 |
| 30. Get comfortable | 0.259 | **0.614** | 0.434 | 0.251 |
| Statistical summary of the scales |  |  |  |  |
| Eigenvalues | 7.209 | 3.414 | 1.983 | 1.716 |
| % of variance explained | 22.528 | 10.669 | 6.196 | 5.362 |

**Supplementary Table S6. Results of exploratory factorial analysis with imposed 6 factors using the 32 coping items of the CHIP questionnaire in early RA (items found in the same dimension are shown in bold).**

| **Items of the original CHIP grouped by subscales** | **Factor 1:** | **Factor 2:** | **Factor 3:** | **Factor 4:** | **Factor 5:** | **Factor 6:** |
| --- | --- | --- | --- | --- | --- | --- |
|  | **Emotional preoccupation** | **Distraction** | **Instrumental** | **Adherence** | **Comforts** | **Rest** |
| Emotional preoccupation |  |  |  |  |  |  |
| 4. Wonder “why me” | **0.647** | 0.148 | 0.297 | 0.205 | 0.295 | 0.447 |
| 8. Feel angry | **0.655** | 0.129 | 0.256 | 0.056 | 0.154 | 0.320 |
| 12. Become frustrated | **0.659** | 0.035 | 0.223 | 0.047 | 0.156 | 0.355 |
| 16. Think about things I can’t do | **0.638** | 0.071 | 0.307 | 0.242 | 0.182 | 0.330 |
| 20. Fantasize about being healthy | **0.599** | 0.218 | 0.196 | 0.217 | 0.319 | 0.246 |
| 24. Wish it hadn’t happed | **0.609** | 0.055 | 0.129 | 0.228 | 0.348 | 0.322 |
| 28. Think about being vulnerable | **0.710** | -0.102 | 0.187 | 0.137 | 0.232 | 0.464 |
| 32. Worry about my health | **0.784** | -0.092 | 0.210 | 0.176 | 0.196 | 0.327 |
| Distraction |  |  |  |  |  |  |
| 1. Think about better times | 0.167 | **0.620** | 0.200 | 0.183 | 0.317 | 0.124 |
| 5. Be with others | 0.076 | **0.610** | 0.291 | 0.110 | 0.171 | 0.229 |
| 9. Daydream | -0.027 | **0.710** | 0.254 | 0.257 | 0.238 | 0.069 |
| 13. Enjoy attention from people | 0.215 | **0.425** | 0.347 | 0.334 | 0.414 | 0.278 |
| 17. Plan for the future | 0.157 | **0.471** | 0.407 | 0.134 | 0.249 | 0.170 |
| 21. Listen to music | 0.086 | **0.359** | 0.298 | 0.143 | 0.331 | 0.178 |
| 25. Invite company | 0.084 | **0.495** | 0.215 | 0.162 | 0.412 | 0.136 |
| 29. Have nice things around | 0.098 | **0.460** | 0.342 | 0.234 | 0.637 | 0.136 |
| Instrumental |  |  |  |  |  |  |
| 3. Find out more information | 0.251 | 0.217 | **0.673** | 0.217 | 0.237 | 0.270 |
| 7. Seek treatment quickly | 0.281 | 0.213 | **0.435** | 0.351 | 0.210 | 0.321 |
| 11. Focus on getting better | 0.111 | 0.362 | **0.594** | 0.342 | 0.203 | 0.155 |
| 15. Learn more | 0.262 | 0.316 | **0.743** | 0.226 | 0.318 | 0.321 |
| 31. Find out about treatment | 0.344 | 0.218 | **0.664** | 0.329 | 0.470 | 0.164 |
| 19. Comply with advice | 0.175 | 0.226 | 0.322 | **0.862** | 0.294 | 0.121 |
| 23. Follow doctor’s advice | 0.185 | 0.228 | 0.321 | **0.823** | 0.414 | 0.097 |
| 27. Take medications on time | 0.145 | 0.152 | 0.186 | **0.543** | 0.318 | 0.117 |
| Palliative |  |  |  |  |  |  |
| 18. Stay warm | 0.280 | 0.333 | 0.211 | 0.340 | **0.411** | 0.319 |
| 22. Make surroundings quiet | 0.286 | 0.277 | 0.302 | 0.311 | **0.616** | 0.417 |
| 26. Stay quiet | 0.337 | 0.008 | 0.111 | 0.263 | **0.511** | 0.407 |
| 30. Get comfortable | 0.202 | 0.347 | 0.314 | 0.392 | **0.742** | 0.236 |
| 2. Stay in bed | 0.368 | 0.047 | 0.194 | -0.039 | 0.217 | **0.567** |
| 6. Rest when tired | 0.350 | 0.169 | 0.265 | 0.194 | 0.288 | **0.708** |
| 10. Sleep | 0.227 | 0.241 | 0.218 | 0.090 | 0.236 | **0.525** |
| 14. Conserve energy | 0.436 | 0.041 | 0.258 | 0.168 | 0.360 | **0.531** |
| Statistical summary of the scales |  |  |  |  |  |  |
| Eigenvalues | 7.209 | 3.414 | 1.983 | 1.716 | 1.538 | 1.244 |
| % of variance explained | 22.528 | 10.669 | 6.196 | 5.362 | 4.807 | 3.886 |
